# Development of Rapid Bioactivity-Expressed Zr-50Ti Alloys by Surface Treatment with Modified Simulated Body Fluid

**DOI:** 10.3390/ijms25126587

**Published:** 2024-06-14

**Authors:** Yuwei Wu, Shigeomi Takai, Takeshi Yabutsuka

**Affiliations:** Graduate School of Energy Science, Kyoto University, Kyoto 606-8501, Japan; wu.yuwei.37w@st.kyoto-u.ac.jp (Y.W.); stakai@energy.kyoto-u.ac.jp (S.T.)

**Keywords:** zirconium–titanium alloy, low crystalline calcium phosphate, apatite-forming ability, bioinoganic trace ions, simulated body fluid, sulfuric acid treatment

## Abstract

Zr-50Ti alloys are promising biomaterials due to their excellent mechanical properties and low magnetic susceptibility. However, Zr-50Ti alloys do not inherently bond well with bone. This study aims to enhance the bioactivity and bonding strength of Zr-50Ti alloys for orthopedic implant materials. Initially, the surface of Zr-50Ti alloys was treated with a sulfuric acid solution to create a microporous structure, increasing surface roughness and area. Subsequently, low crystalline calcium phosphate (L-CaP) precipitation was controlled by adding Mg^2+^ and/or CO_3_^2−^ ions in modified simulated body fluid (m-SBF). The treated Zr-50Ti alloys were then subjected to cold isostatic pressing to force m-SBF into the micropores, followed by incubation to allow L-CaP formation. The apatite-forming process was tested in simulated body fluid (SBF). The results demonstrated that the incorporation of Mg^2+^ and/or CO_3_^2−^ ions enabled the L-CaP to cover the entire surface of Zr-50Ti alloys within only one day. After short-term soaking in SBF, the L-CaP layer, modulated by Mg^2+^ and/or CO_3_^2−^ ions, formed a uniform hydroxyapatite (HA) coating on the surface of the Zr-50Ti alloys, showing potential for optimized bone integration. After soaking in SBF for 14 days, the bonding strength between the apatite layer and alloy has the potential to meet the orthopedic application requirement of 22 MPa. This study demonstrates an effective method to enhance the bioactivity and bonding strength of Zr-50Ti alloys for orthopedic applications.

## 1. Introduction

Titanium (Ti) alloys are widely used in dentistry and orthopedics due to their excellent mechanical properties, corrosion resistance, and biocompatibility. Zirconium (Zr) shares comparable physical and chemical properties as the same group element as Ti. Additionally, Zr exhibits significantly lower magnetic susceptibility, approximately one-third that of Ti [1]. During diagnostic procedures involving magnetic apparatus, such as magnetic resonance imaging (MRI), metallic material with high magnetic susceptibility can cause artifacts, complicating the diagnosis. Moreover, it is reported that the Zr-Ti and Ti alloy implants have similar bone-implant contact (BIC). However, the removal torque (RTQ) of Zr-Ti alloy implants is higher than that of Ti alloys [2]. Zr-45Ti alloys have been identified as promising biomedical devices due to their excellent electrochemical stability and biocompatibility [3]. Zr-50Ti alloys possess a lower elastic modulus (approximately 90 MPa [4]) than pure Zr and Ti. Their elastic modulus is close to that of Zr-40Ti, the lowest among Zr-Ti binary alloys. The apatite-forming ability of Zr-Ti alloys significantly decreases when the Zr content exceeds 60 atom% [5]. These properties suggest that Zr-50Ti alloys could be a promising biomaterial.

Despite these advantages, Zr-50Ti, being a metallic material, cannot directly bond with bone. Surface modification is required to impart bioactivity to Zr-50Ti alloys. Various methods can enhance the bioactivity of metallic materials, including physical methods such as plasma spraying [6], and electrophoretic deposition [7]. However, chemical methods, such as the sol-gel method [8] and alkali-heat treatment [9], have been developed due to the risk of peeling and the high costs associated with the physical processes.

Bioactivity can be evaluated by simulated body fluid (SBF), which mimics the inorganic composition of human blood plasma [10]. When the temperature and pH of SBF are increased, fine particles of amorphous calcium phosphate precipitate in the solution through homogeneous nucleation. Yao et al. found that these particles, termed apatite nuclei (AN), were highly bioactive and could induce apatite formation in SBF [11]. AN is a promising material for imparting bioactivity to various substrates through surface treatment. We have fabricated novel biomaterials with high bioactivity and diverse mechanical properties by precipitating AN on acid-treated porous titanium alloys, zirconium alloys, and PEEK [12,13,14].

On the other hand, despite the bioactivity imparted to the substrate material, the apatite formed in SBF bonds with the metallic substrate with weak strength, leading to a risk of the apatite coating peeling off. Miyazaki et al. reported that although apatite can successfully precipitate on the Zr-50Ti alloys, the adhesive strength between apatite and the surface of Zr-50Ti alloys is insufficient [15]. According to the F1147-05 regulation of the American Society for Testing and Materials (ASTM) for calcium phosphate and metallic coatings, a minimum adhesion strength of 22 MPa is suitable for orthopedic applications. The bonding can be enhanced by mechanical interlocking; therefore, an additional surface treatment is necessary before the bioactivity treatment.

In this study, the surface morphology of Zr-50Ti alloys was modified using a sulfuric acid solution to form micropores on the surface, which can enhance the bonding ability between the apatite coating and Zr-50Ti substrates. Based on our previous research, the SBF was modified by removing interfering ions and enhancing the concentration of necessary ions, creating a modified-SBF (m-SBF). Low crystalline calcium phosphate (L-CaP) was precipitated on the surface of micropore-formed Zr-50Ti alloys in the supersaturated m-SBF. After this process, bioactivity was imparted to the Zr-50Ti alloys, and it was evaluated by soaking the L-CaP-precipitated Zr-50Ti alloys in the SBF for 1, 3, 7, and 14 days and observing apatite-forming performance. Unlike our previous studies, our method achieved rapid and uniform L-CaP precipitation without the formation of large, uneven apatite aggregates, and the bonding strength between the apatite and substrate has the potential to meet the requirement of ASTM F1147-05.

## 2. Results

### 2.1. Surface Roughness of Zr-50Ti Alloys Treated by Various Volume Ratio Sulfuric Acid Solution

**Figure 1:**
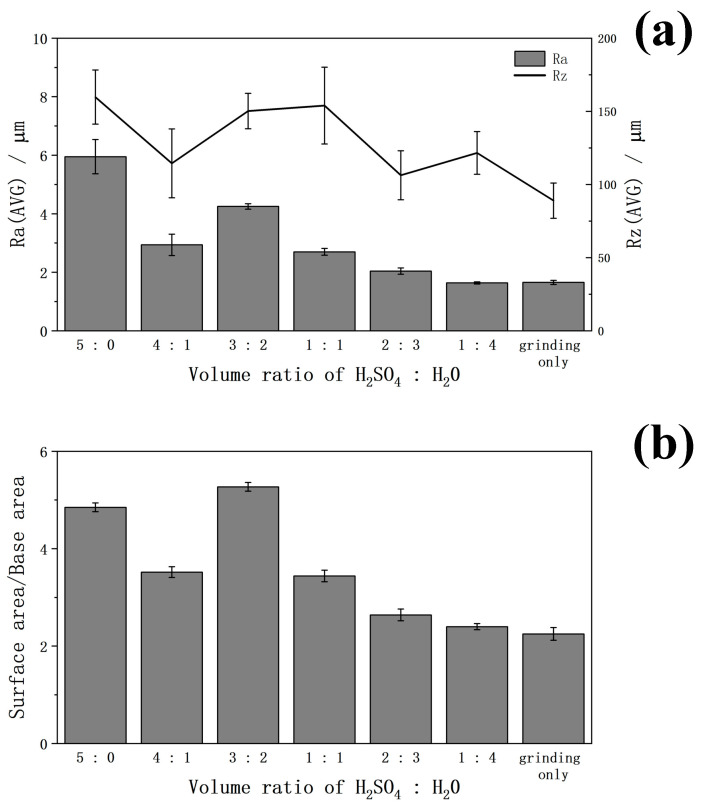
The (**a**) surface roughness and (**b**) surface area/base area of Zr-50Ti alloys treated by the various volume ratios (95wt% H_2_SO_4_:H_2_O) sulfuric acid solution at 70 °C for 3 h.

In Figure 1a, the surface roughness of Zr-50Ti alloys treated by the various volume ratio sulfuric acid solutions is shown. The Ra represents the average roughness, while the Rz indicates the maximum peak-to-valley height of the profile. According to these data, the surface roughness of Zr-50Ti alloys was enhanced by the sulfuric acid solution except for the volume ratio of 1:4, which may be due to the acidity being too weak to react. Although the Zr-50Ti alloys treated with 5:0 sulfuric acid solution had the highest surface roughness, the standard deviation is very high, likely due to uneven passivation. The relatively low surface roughness of the volume ratio of 4:1 is attributed to the high dissolution of Zr-50Ti alloys in this ratio of sulfuric acid solution. In comparison, the 3:2 volume ratio of the sulfuric acid solution resulted in Zr-50Ti alloys with relatively high surface roughness, as well as a very low standard deviation and the highest ratio of surface area/base area as shown in Figure 1b. Therefore, the volume ratio of 3:2 is considered the most effective volume ratio to treat the Zr-50Ti alloys.

### 2.2. Pore Formation by 3:2 Sulfuric Acid Solution Treatment

**Figure 2:**
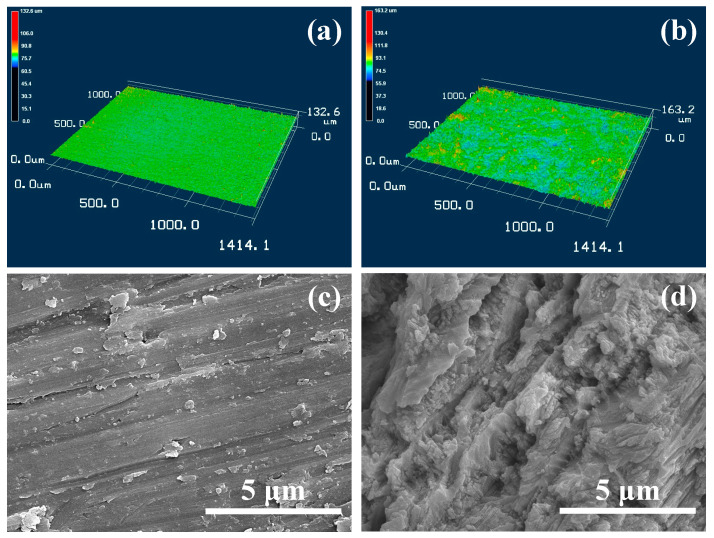
The 3D surface morphology of Zr-50Ti alloys (**a**) before and (**b**) after sulfuric solution treatment (3:2). The SEM surface morphology of Zr-50Ti alloys (**c**) before and (**d**) after sulfuric solution treatment (3:2).

The 3D and SEM surface morphologies of Zr-50Ti alloys before and after the 3:2 volume ratio sulfuric acid solution treatment are shown in Figure 2. Before the sulfuric acid treatment, the Zr-50Ti alloys ground by the abrasive paper exhibited a relatively smooth surface. After sulfuric acid treatment, vertical erosion was observed on the surface of the Zr-50Ti alloys, which improved the overall surface roughness and surface area by changing the surface morphology.

**Figure 3:**
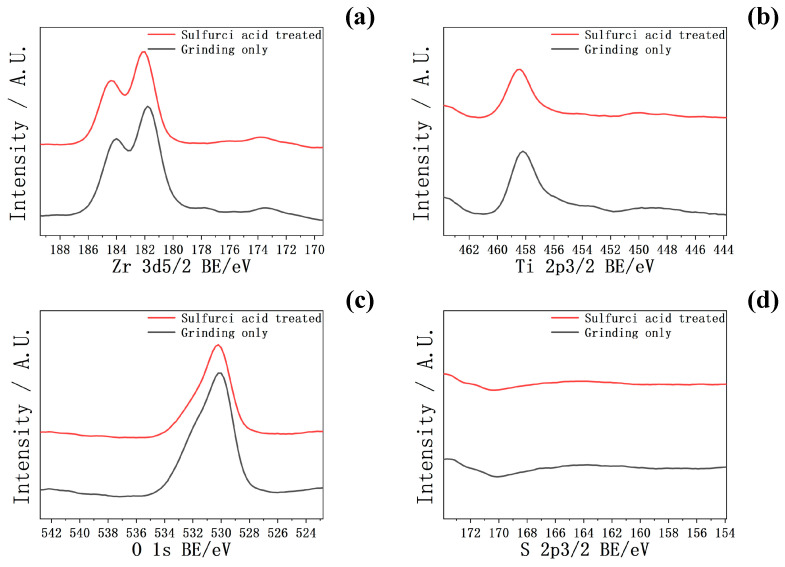
The XPS of (**a**) Zr, (**b**) Ti, (**c**) O, and (**d**) S of Zr-50Ti alloys before and after sulfuric acid solution (3:2) treatment.

In Figure 3, the XPS spectra of Zr-50Ti alloys before and after the 3:2 volume ratio sulfuric acid treatment are shown. After sulfuric acid treatment, the intensities of Zr, Ti, O, and S in the XPS spectra were similar to those before the treatment.

**Figure 4:**
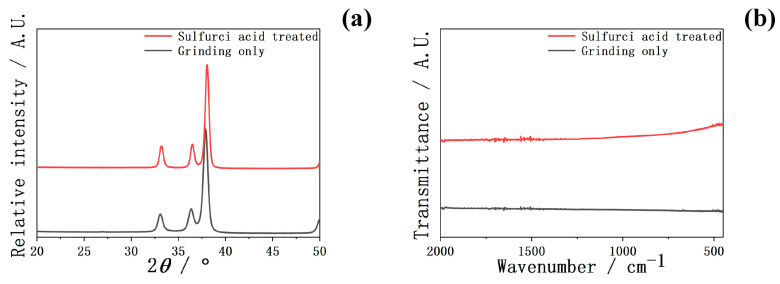
The (**a**) TF-XRD and (**b**) FTIR of Zr-50Ti alloys before and after sulfuric acid solution (3:2) treatment.

In Figure 4, the XRD and FTIR spectra of Zr-50Ti alloys before and after the 3:2 volume ratio sulfuric acid treatment are shown. The intensities of XRD and FTIR spectra were also comparable before and after sulfuric acid treatment. These findings suggest that the by-products of the sulfuric acid treatment were removed after 30 min of ultrasonic cleaning.

### 2.3. Surface Morphology after Soaking in Various m-SBF

**Figure 5:**
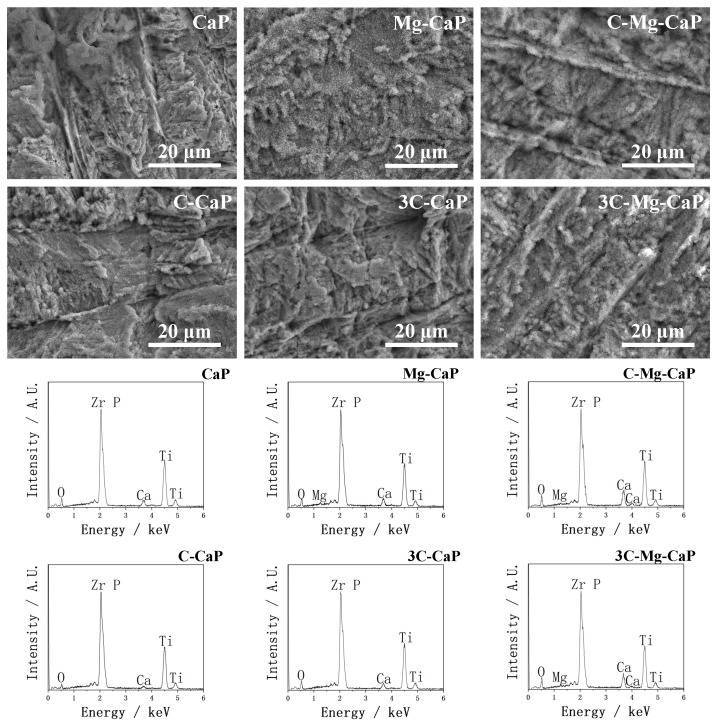
SEM images and EDX spectra of various m-SBFs-treated Zr-50Ti alloys.

In Figure 5, the SEM and EDX images show the surface morphology and surface elements of various m-SBFs-treated Zr-50Ti alloys, respectively. In the SEM images, the uncovered surface of Zr-50Ti alloys was observed only in the group CaP, with only a few surface areas covered by gigantic L-CaP precipitates, indicating extremely uneven calcium phosphate precipitation in CaP m-SBF. The other groups of Zr-50Ti alloys were fully and relatively uniformly covered by L-CaP. In the EDX analysis, the P overlapped with Zr; therefore, only Ca was clearly detected in all groups. In groups Mg-CaP, C-Mg-CaP, and 3C-Mg-CaP, the Mg was also detected.

**Figure 6:**
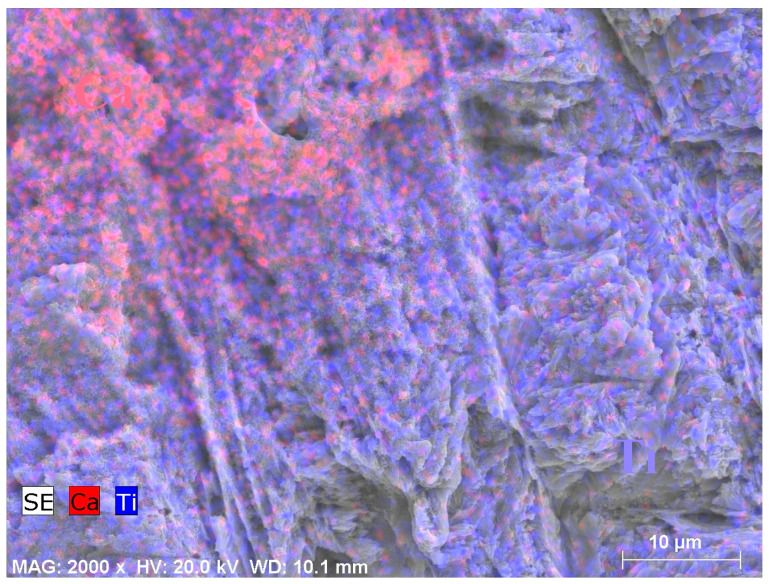
EDX mapping with SEM image of CaP m-SBF-treated Zr-50Ti alloys.

In Figure 6, due to the uneven precipitation of group CaP, EDX mapping was also conducted. The red area represented Ca, and the purple area represented Ti, which clearly showed many areas without calcium phosphate precipitates.

**Figure 7:**
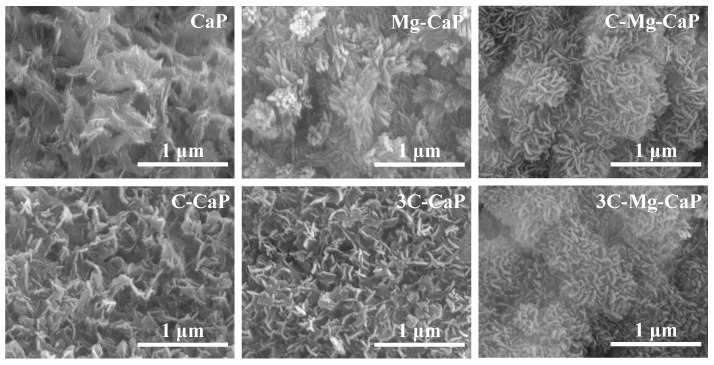
Magnification of SEM images from the center of the corresponding SEM images in Figure 5.

In Figure 7, the SEM images which were magnified from the center of the corresponding SEM of Figure 5 images were shown. In group CaP, the largest L-CaP further confirmed the uneven precipitation. In groups C-CaP and 3C-CaP, the incorporation of the CO_3_^2−^ ion made the L-CaP smaller than in group CaP m-SBF. In group Mg-CaP, the incorporation of Mg^2+^ ion altered the shape of L-CaP compared to group CaP, C-CaP, and 3C-CaP. In groups C-Mg-CaP and 3C-Mg-CaP, the incorporation of both Mg^2+^ and CO_3_^2−^ ions made the L-CaP even smaller than the incorporation of only Mg^2+^ or CO_3_^2−^ ion. Additionally, the enhancement of CO_3_^2−^ ion concentration did not show a significant change in the crystal morphology.

### 2.4. Surface Morphology after Soaking in Various m-SBF

**Figure 8:**
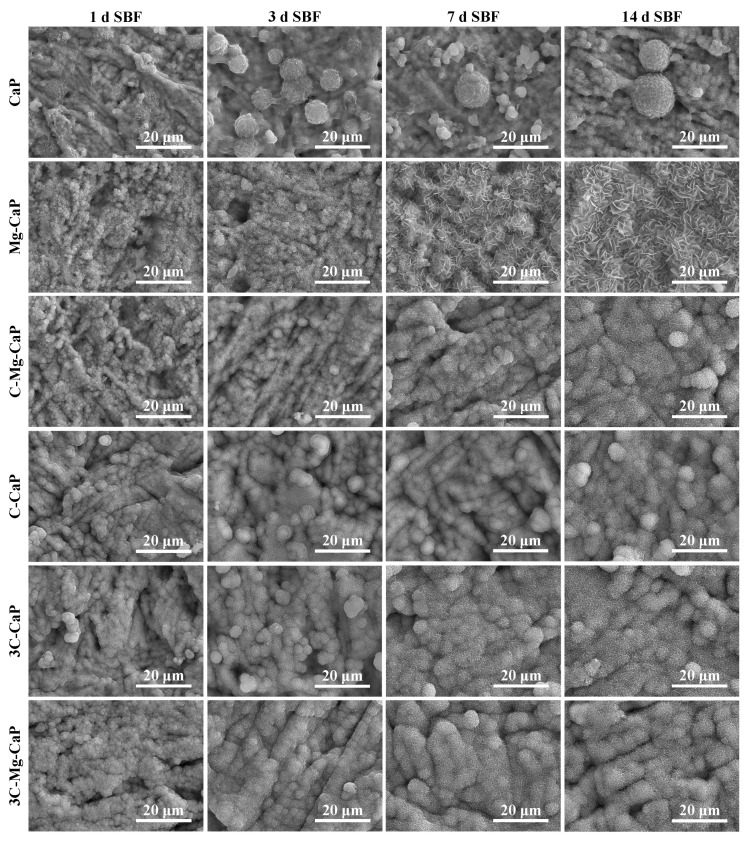
SEM images of SBF-soaked Zr-50Ti alloys after 1, 3, 7, and 14 days.

The surface morphology of the growth status of various L-CaPs on Zr-50Ti alloys after soaking in SBF for 1, 3, 7, and 14 days is shown in Figure 8. In group CaP, due to the uneven precipitation of L-CaP during m-SBF treatment, the apatite grown from the L-CaP was also uneven after soaking in SBF, forming the gigantic ball-like apatite aggregates. Eventually, the uncovered areas of group CaP disappeared in 3 d SBF-soaked samples. In comparison, the entire Zr-50Ti alloy surface could be covered during the m-SBF treatment in the other groups. In those groups, except for group Mg-CaP, although the apatite grown from L-CaP showed different surface morphology in Figure 5 and Figure 8, after 3 d of SBF soaking, the crystal morphology of apatite became similar. In group Mg-CaP, due to the lack of CO_3_^2−^ ion, the surface morphology was different from the others, especially after 7 d of SBF soaking.

**Figure 9:**
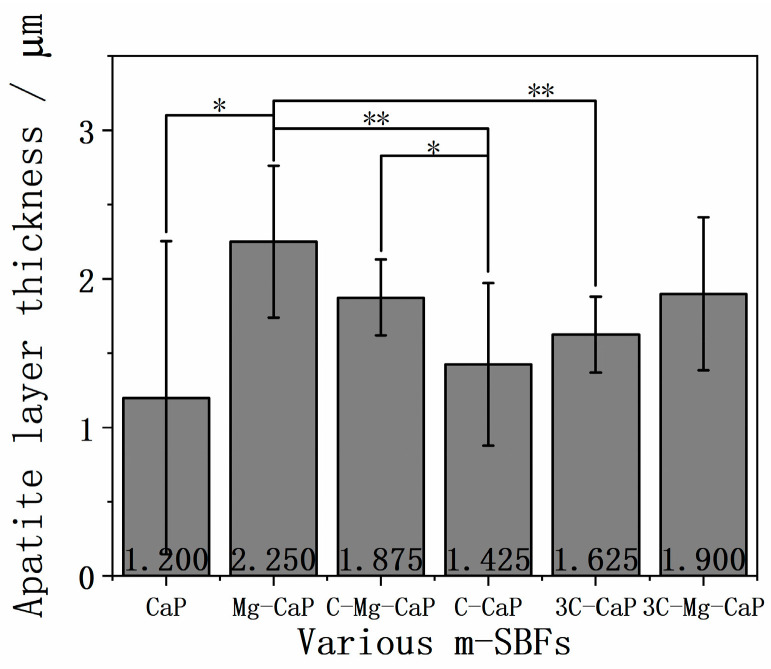
The thickness of L-CaP of various m-SBFs-treated Zr-50Ti alloys (n = 10). The symbol “**” indicates *p* < 0.01, “*” indicates *p* < 0.05, and the absence of a symbol indicates *p* > 0.05, as determined by the Student’s *t*-test.

In Figure 9, the thickness of the L-CaP formed in various m-SBFs is shown. In group CaP, many uncovered areas were observed, and the L-CaP was much larger than in the other groups, as shown in Figure 5, leading to an incredibly large standard deviation. This graph confirmed that the Mg^2+^ ion can increase the thickness of the L-CaP layer. Although the CO_3_^2−^ ion also enhanced the thickness of L-CaP, there was no significance. Moreover, the incorporation of Mg^2+^ or CO_3_^2−^ ion resulted in a much lower standard deviation.

**Figure 10:**
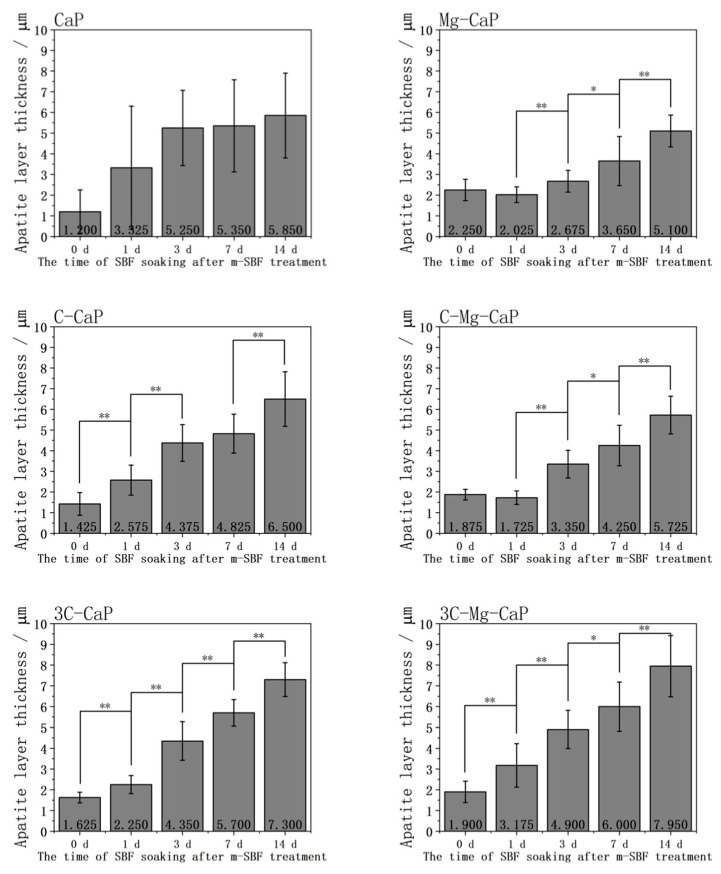
The thickness of L-CaP of various m-SBFs-treated Zr-50Ti alloys and apatite of 1, 3, 7 and 14 days SBF-soaked Zr-50Ti alloys (n = 10). The symbol “**” indicates *p* < 0.01, “*” indicates *p* < 0.05, and the absence of a symbol indicates *p* > 0.05, as determined by the Student’s *t*-test. Comparisons are limited to adjacent time points.

In Figure 10, the thickness of the various L-CaP layers before and after soaking in SBF are shown. In group CaP, the uneven L-CaP grew rapidly but still exhibited a great standard deviation after soaking in SBF. In the other groups, the incorporation of Mg^2+^ or CO_3_^2−^ ion resulted in apatite grown from the L-CaP with a significantly lower standard deviation. In group Mg-CaP, a thick L-CaP layer formed on the surface; however, it is difficult to grow in thickness after 1 d of SBF soaking despite changes in the surface morphology, as shown in Figure 5 and Figure 8. In group C-CaP, the L-CaP grew rapidly after soaking in SBF; however, the L-CaP layer was thinner compared with group Mg-CaP and enhancing the CO_3_^2−^ ion did not resolve this issue. By incorporating both Mg^2+^ and CO_3_^2−^ ions, in group 3C-Mg-CaP, a relatively thick L-CaP layer was formed which could grow thicker after 1 d of SBF soaking.

**Figure 11:**
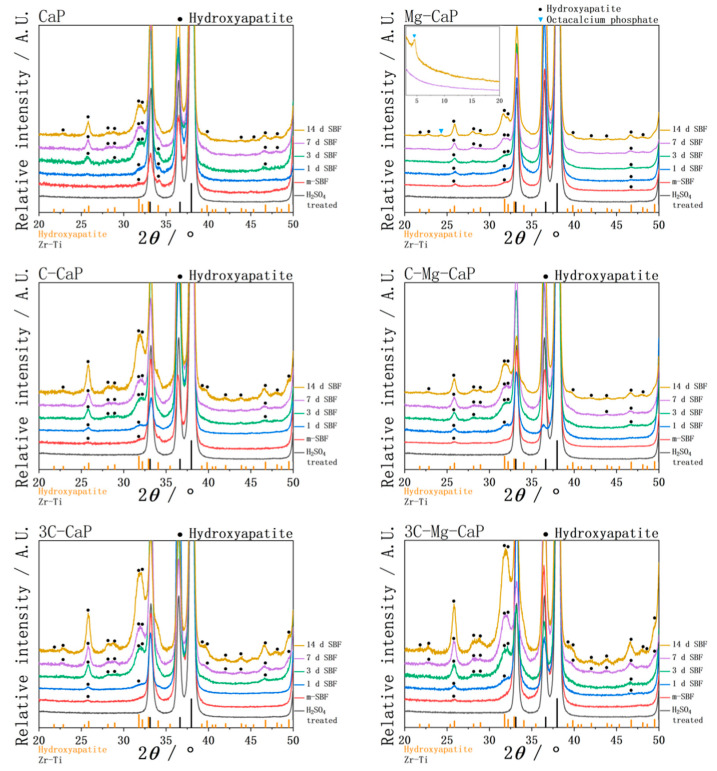
The TF-XRD of L-CaP of various m-SBFs-treated Zr-50Ti alloys and apatite of 1, 3, 7 and 14 days SBF-soaked Zr-50Ti alloys.

In Figure 11, the TF-XRD patterns of various L-CaPs before and after soaking in SBF are shown. The characteristic peaks around 26, 32, and 34 degrees indicate that all L-CaPs crystallized during m-SBF treatment. However, these degrees were common peaks of calcium phosphate, making it difficult to determine the specific types of calcium phosphate present in the L-CaPs. After SBF soaking, the characteristic peaks suggest that all L-CaPs transformed into hydroxyapatite in SBF, despite the different surface morphology as shown in Figure 5. Additionally, the intensity of XRD peaks was highly correlated with the thickness; greater thickness resulted in higher intensity of the characteristic peaks. Only the 14 d SBF-soaked sample in group Mg-CaP exhibited a peak around 24 degrees, which does not correspond to hydroxyapatite. In a wider range XRD characterization, a sharp peak around 4 degrees suggested that the 24-degree peak belongs to octacalcium phosphate (OCP). Thus, after 14 d of SBF soaking in group Mg-CaP, both hydroxyapatite and OCP were formed.

**Figure 12:**
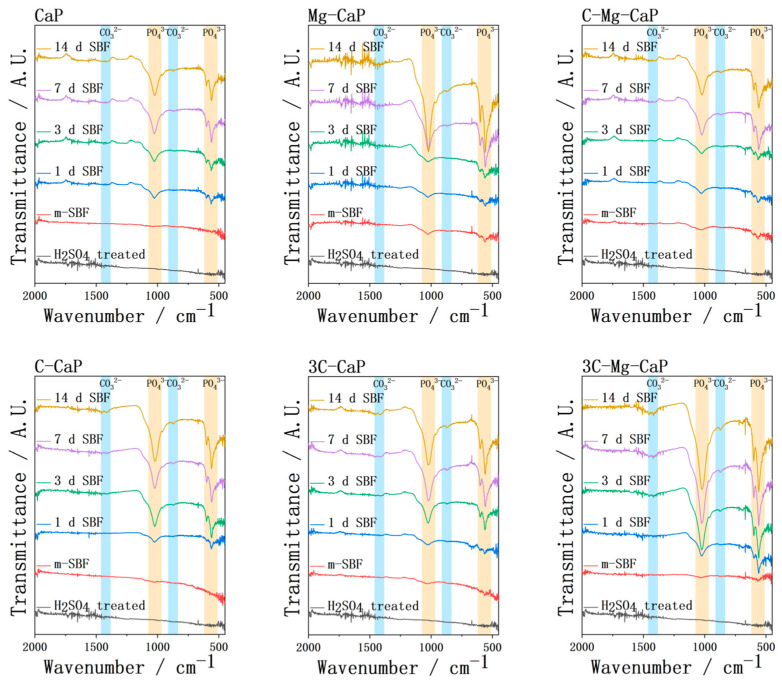
The FTIR of L-CaP of various m-SBFs-treated Zr-50Ti alloys and apatite of 1, 3, 7 and 14 days SBF-soaked Zr-50Ti alloys.

The FTIR spectra of various L-CaPs before and after soaking in SBF are shown in Figure 12. After m-SBF treatment, the absorption bands of PO_4_^3−^ were detected in all groups except for group CaP. However, due to uneven precipitation, the PO_4_^3−^ bands varied greatly, resulting in both weak and strong bands being detected in group CaP. The intensity of FTIR spectra was also highly correlated with the thickness; therefore, the group Mg-CaP exhibited the most pronounced bands due to the thick L-CaP layer. Additionally, in group Mg-CaP, the formation of the OCP, which has higher phosphate content than hydroxyapatite, led to a sudden intensification of PO_4_^3−^ absorption bands after 7 d of SBF soaking. CO_3_^2−^ bands were also detected in all groups, even in group CaP and group Mg-CaP, due to the presence of CO_3_^2−^ ion in the SBF. The higher the CO_3_^2−^ concentration in m-SBF, the faster the formation of detachable absorption bands. However, even in the group 3C-CaP and 3C-Mg-CaP, detachable CO_3_^2−^ bands were formed after 3 d of SBF soaking, while the PO_4_^3−^ bands remained strong, suggesting that only a small amount of CO_3_^2−^ was incorporated into the apatite structure.

**Figure 13:**
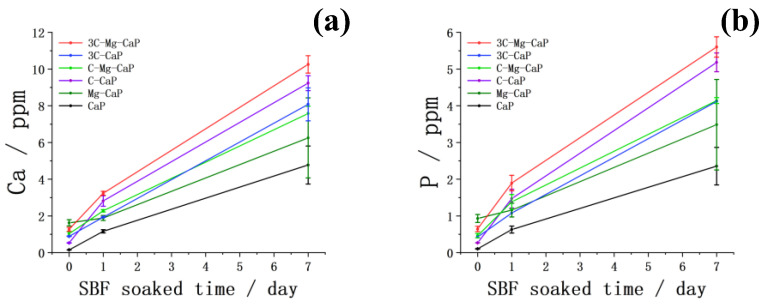
The (**a**) calcium and (**b**) phosphorous concentration of L-CaP on m-SBF-treated Zr-50Ti alloys, and apatite on 1 d and 7 d SBF-soaked Zr-50Ti alloys, which dissolved in 50 mL 1 M HNO_3_, respectively (n ≥ 3).

The L-CaP on m-SBF-treated Zr-50Ti alloys, and apatite on 1 d and 7 d SBF-soaked Zr-50Ti alloys, were dissolved in 50 mL 1 M HNO_3_, respectively. The Ca and P concentrations characterized by ICP are shown in Figure 13. The Ca and P concentrations of group CaP show less precipitation of calcium phosphate without the incorporation of any ions during both m-SBF treatment and SBF soaking periods. The incorporation of Mg^2+^ ion increased the precipitation of L-CaP in m-SBF treatment; however, it did not significantly enhance the apatite-forming ability after soaking in SBF. On the other hand, the incorporation of the CO_3_^2−^ ion into the L-CaP in the m-SBF treatment, although it only slightly increased the amount of L-CaP precipitation, resulted in much faster growth of apatite after soaking in SBF. Finally, the 3C-Mg-CaP m-SBF led to well-precipitated L-CaP during m-SBF treatment and the fastest growth after soaking in SBF compared to the other five groups.

In Table 1, the Ca/P and (Ca + Mg)/P of the various L-CaPs are shown. The L-CaP formed in CaP m-SBF has the lowest Ca/P ratio. Both the incorporation of Mg^2+^ or CO_3_^2−^ ion can enhance the Ca/P ratio, with CO_3_^2−^ ion being more effective in enhancing the Ca/P ratio.

**Figure 14:**
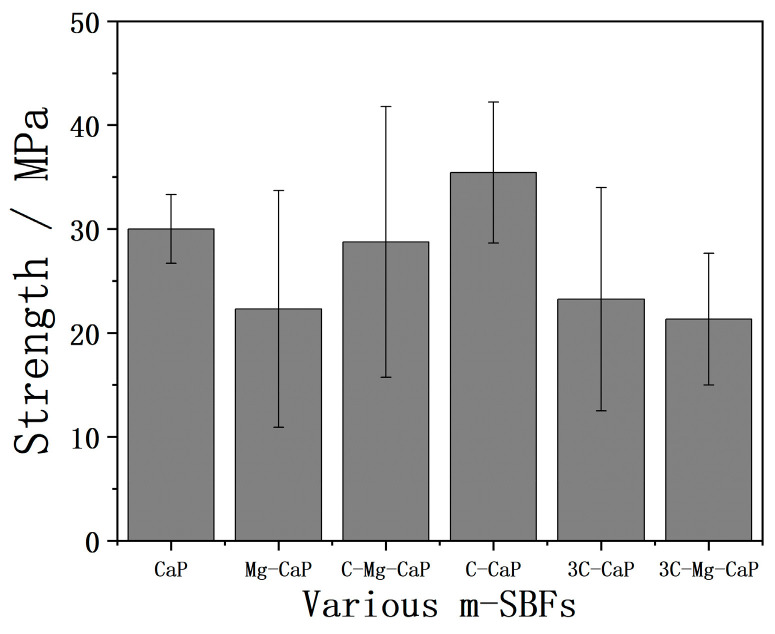
The obtained strength of 14 d SBF-soaked Zr-50Ti alloys by modified ASTM C-633 method (n ≥ 3).

**Figure 15:**
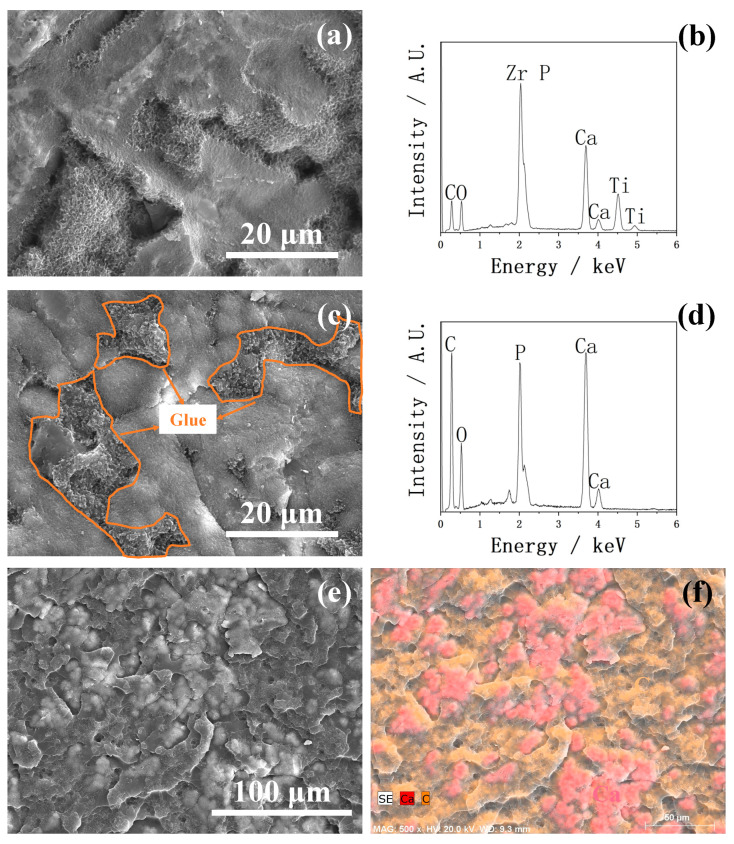
The SEM images of fracture areas of the 14 d SBF-soaked Zr-50Ti alloys in group 3C-Mg-CaP. (**a**) Zr-50Ti side of the fracture area. (**b**) EDX of (**a**). (**c**) Jig side of the fracture area. (**d**) EDX of (**c**). (**e**) Jig side with lower magnification. (**f**) EDX mapping of (**e**), red is calcium which represents apatite, and orange is carbon which represents the glue.

The strength of 14 d SBF-soaked Zr-50Ti alloys, tested by the modified ASTM C-633 method, is shown in Figure 14. The average strength of all groups was over 21 MPa but with high standard deviations. The SEM images of the fracture area of 14 d SBF-soaked Zr-50Ti alloys in group 3C-Mg-CaP are shown in Figure 15. In Figure 15a, both the fractured apatite and the intact apatite were observed on the Zr-50Ti side, suggesting that the fracture was incomplete. In Figure 15b, the strong Ca peak indicates that a substantial amount of apatite remained on the Zr-50Ti alloy. Figure 15c,d also support the suspicion of incomplete fracture, as the glue without apatite coverage was observed on the jig side. The low magnification SEM and EDX mapping of the jig side as shown in Figure 15e,f, respectively, suggest that only a small amount of apatite peeled off from the apatite layer. It can be concluded that the fracture occurred primarily between the apatite layer and the glue layer, with only a small amount of apatite peeled off. As a result, the actual adhesive strength between the apatite and Zr-50Ti alloys would be higher than these data.

## 3. Materials and Methods

### 3.1. Materials

The Zr-50Ti alloy with an atomic ratio of Zr:Ti = 1:1 had a plate size of 15 × 10 × 2 mm^3^ (E-Metals, Osaka, Japan). The Zr-50Ti alloys were ground using #1200 and #2000 silicon carbide (SiC) abrasive papers with a rotary wet grinder. Subsequently, the ground Zr-50Ti alloys were treated by ultrasonic cleaning in acetone for 10 min.

### 3.2. Micropore Formation by Sulfuric Acid Treatment

Sulfuric acid (FUJIFILM Wako Pure Chemicals, 95 wt%) was mixed with ultrapure water at volume ratios of 5:0, 4:1, 3:2, 1:1, 2:3, and 1:4. The Zr-50Ti alloys were immersed in these sulfuric acid solutions and then treated with water bathing at 70 °C for 3 h. Following this, the sulfuric acid-treated Zr-50Ti alloys were treated by ultrasonic cleaning in ultrapure water for 30 min and were air-dried for 1 day. This process resulted in the formation of micropores on the surface.

### 3.3. Measurement of the Zr-50Ti Alloys before and after Sulfuric Acid Treatment

The surface roughness and surface area of Zr-50Ti alloys treated by various volume ratio sulfuric acid solutions were characterized by color 3D laser microscopy (VK-9700, Keyence, Osaka, Japan) (JIS B0601: 2001). The surface morphology of the Zr-50Ti alloys was characterized by thin film X-ray diffraction (TF-XRD, X’Pert PRO, PANalytical, Almelo, The Netherlands), using CuKα X-ray with tube voltage and current of 45 kV and 40 mA. Additionally, scanning electron microscopy (SEM, SU6600, Hitachi High-Tech, Tokyo, Japan), energy dispersive X-ray spectroscopy (EDX, XFlash 5010, Bruker, Billerica, MA, USA), Fourier transform infrared spectroscopy with diamond ATR methods (FTIR, FT/IR-4700, JASCO, Tokyo, Japan) with the number of scans at 100 and resolution at 2 cm^−1^, and MgKα X-ray photoelectron spectroscopy (XPS, JPS-9030, JEOL, Tokyo, Japan) at 12 kV and 10 mA were also conducted.

### 3.4. Preparation of SBF and m-SBF

Kokubo et al. reported that the apatite-forming ability can be predicted in SBF environment [10]. As a result, we used the SBF to evaluate the bioactivity of the Zr-50Ti alloys obtained in this study. Reagent-grade NaCl (FUJIFILM Wako Pure Chemicals, 99.5%), NaHCO_3_ (Hayashi Pure Chemicals, 99.5%~), KCl (Hayashi Pure Chemicals, 99.5%), K_2_HPO_4_·3H_2_O (Nacalai Tesque, 99.0%), MgCl_2_·6H_2_O (Hayashi Pure Chemicals, 98.0%), CaCl_2_ (FUJIFILM Wako Pure Chemicals, 95.0%) and Na_2_SO_4_ (Hayashi Pure Chemicals, 99.0%) were dissolved in ultrapure water. The pH value was adjusted to 7.40 with tris(hydroxymethyl)aminomethane ((CH_2_OH)_3_CNH_2_, FUJIFILM Wako Pure Chemicals, 95.0%) and 1 M HCl (FUJIFILM Wako Pure Chemicals) at 36.5 °C by the method certified in ISO 23317 [16]. After this process, an aqueous solution with inorganic ions concertation similar to human blood plasma was obtained.

The previous m-SBF was modified from SBF by removing interfering ions, specifically NaCl, KCl, and Na_2_SO_4_. The pH was then adjusted to 8.20 by dissolving (CH_2_OH)_3_CNH_2_ at 25.0 °C, which favors the precipitation of AN [14]. In this study, before adjusting the pH, we customized the addition of MgCl_2_·6H_2_O and NaHCO_3_ to investigate the role of Mg^2+^ and CO_3_^2−^ ions in the precipitation of L-CaP and the formation of the apatite after soaking in SBF. The ion concentration of human blood plasma, SBF, and various m-SBFs are shown in Table 2.

### 3.5. m-SBF Treatment

To ensure the various types of m-SBF infiltrated into the micropores, the Zr-50Ti alloys soaked in m-SBF were treated by cold isostatic pressing (CIP, Kobelco, Kobe, Japan) at 100 MPa for 1 h. Then, the CIP-treated Zr-50Ti alloys were soaked in the same m-SBF and incubated at 70 °C for 24 h to facilitate the formation of L-CaP in the micropores. After this process, the Zr-50Ti alloys acquired L-CaP.

### 3.6. The Evaluation of Bioactivity

The bioactivity, also referred to as the apatite-forming ability, was tested by soaking the bioactive substrates in SBF for 1, 3, 7, and 14 days at 36.5 °C. The Zr-50Ti alloys were characterized by TF-XRD, SEM, EDX, and FTIR.

### 3.7. Measurement of the Thickness of the Apatite Layer

The m-SBF-treated (after CIP-treated and m-SBF-treated, hereafter m-SBF-treated) and SBF-soaked (after CIP-treated, m-SBF-treated and SBF-soaked, hereafter SBF-soaked) Zr-50Ti alloys were immersed in epoxy resin (23 mL Epok 812, 15 mL DDSA, 12 mL MNA, 0.75 mL DMP-30, Okenshoji, Tokyo, Japan) and incubated at 50 °C for 3 days. After the epoxy resin solidified, the Zr-50Ti alloys were cut in the middle. The cross-sections of the Zr-50Ti alloys were observed by SEM and EDX line analysis.

### 3.8. Measurement of the Element Composition of Apatite by Inductively Coupled Plasma Atomic Emission Spectroscopy (ICP)

The m-SBF-treated Zr-50Ti alloys or SBF-soaked Zr-50Ti alloys were soaked in 50 mL of 1 M HNO_3_ (FUJIFILM Wako Pure Chemicals) for 1 and 7 days, respectively. The solution was sealed at room temperature for 3 days to dissolve the apatite. The Ca, P, and Mg concentrations were measured by inductively coupled plasma atomic emission spectroscopy (ICP, ICPS-7510, Shimadzu, Kyoto, Japan). The standard calibration solution was prepared by using a calcium standard solution (1000 mg/L, FUJIFILM Wako Pure Chemicals), a phosphorus standard solution (1000 mg/L, Wako Pure Chemicals), and a magnesium standard solution (1000 mg/L, FUJIFILM Wako Pure Chemicals) dissolved in ultrapure water.

### 3.9. Measurement of the Mechanical Strength

The mechanical strength of samples soaked in SBF for 14 days was measured by the modified ASTM C-633 method [17]. Both sides of the apatite-covered Zr-50Ti alloy were attached to stainless steel jigs (10 × 10 mm^2^) with Araldite 2000 glue (Huntsman, The Woodlands, TX, USA). The tensile load was applied at a crosshead speed of 1 mm/min by a universal testing machine (Autograph AGS-H, Shimadzu, Japan) until the jig and substrate were separated. Fractures on both the jig side and the substrate side were observed by SEM and EDX.

## 4. Discussion

Ban et al. [18] reported the surface of commercially pure Ti modified by sulfuric acid etching. Considering that Zr belongs to the same group element as Ti, its reaction with sulfuric acid may be similar to that of Ti. Ban et al. suggested that three reactions occur, with reaction (2) being dominant. In XRD, the TiH_2_ should have a strong peak at 40.9 degrees.

TiO_2_ + 2H_2_SO_4_ → Ti(SO_4_)_2_ + 2H_2_O (1)

Ti + 2H_2_SO_4_ → Ti(SO_4_)_2_ + 2H_2_ (2)

Ti + H_2_ → TiH_2_ (3)

However, in Figure 4a, the XRD pattern of Zr-50Ti alloys after sulfuric acid treatment did not exhibit that peak. Additionally, in Figure 3d, the absence of the S peak in the XPS spectrum also indicated no Ti(SO_4_)_2_ remaining on the surface of Zr-50Ti alloys. It is considered that after 30 min ultrasonic cleaning, all of the sulfides had been washed away. As a result, it can be concluded that the by-products of Zr-50Ti alloys in the sulfuric acid reaction can be washed out.

The transformation of amorphous calcium phosphate (ACP) into crystallized apatite has been extensively researched. The crystallization of ACP is less than 1 h [19,20,21] and 2 h [22] has been reported. In this study, soaking in various supersaturated m-SBFs at 70 °C for 1 day ensured that the L-CaP started crystallizing, as shown in Figure 11.

There is also extensive research on the effect of Mg^2+^ and CO_3_^2−^ ions on ACP, both Mg^2+^ [19,20,21,22,23] and CO_3_^2−^ [19] ions can stabilize ACP and retard its crystallization into apatite. Termine et al. [19] particularly claim that the stabilizing strength of the Mg^2+^ ion is much stronger than that of CO_3_^2−^ ion. The comparison in Figure 7 may support this. Without any doping, the group CaP demonstrates the formation of gigantic apatite crystals, the doping of CO_3_^2−^ ion allowed the formation of smaller flake-like apatite, while the incorporation of Mg^2+^ ion results in the formation of even smaller needle-like apatite.

Furthermore, Mg^2+^ and CO_3_^2−^ ions can enhance the amount and uniformity of L-CaP precipitation, and increase the Ca/P ratio of L-CaP, as shown in data from Figure 10 and Figure 13, and Table 1. Consequently, the apatite induced by various L-CaPs exhibited significant differences in growth performance in SBF. The more intensively ACP is stabilized, the greater the amount of calcium phosphate precipitated. It is reasonable to speculate that by inhibiting crystal growth through the incorporation of Mg^2+^ or CO_3_^2−^ ions, the supersaturated calcium phosphate will not aggregate on the already precipitated L-CaP, instead, it can help the calcium phosphate to form new precipitates independently. Therefore, the precipitation of L-CaPs, except group CaP, can cover the entire surface of the Zr-50Ti alloys within 1 d of m-SBF treatment.

After 14 d of SBF soaking in group Mg-CaP, OCP formed together with the hydroxyapatite. The formation of ACP or apatite causes a decrease in pH [21,24], and De Rooij et al. [25] suggest that at pH > 7, hydroxyapatite forms preferentially, while at pH < 7, OCP tends to form. Therefore, in this study, the incorporated CO_3_^2−^ ion can serve as a buffer to maintain the pH. Consequently, in the L-CaP only with Mg^2+^ ion, the pH decreases rapidly caused by the apatite formation, which may lead to the formation of OCP.

Müller et al. [26] reported that the CO_3_^2−^ contained apatite is more likely to form than stoichiometric hydroxyapatite. The incorporation of a Mg^2+^ or CO_3_^2−^ ion into L-CaP significantly enhanced the growth speed during SBF soaking as shown in Figure 10 and Figure 13. In Table 1, the data showed the Ca/P ratio was 1.5 in group CaP, which indicated that tricalcium phosphate (TCP) may tend to form at this ratio. The incorporation of the Mg^2+^ ion makes the Ca/P ratio of L-CaP close to 1.67, which is the Ca/P ratio of hydroxyapatite. By incorporating the CO_3_^2−^ ion, Ca-rich L-CaP with high apatite-forming potential was formed during the m-SBF treatment. This explains the CO_3_^2−^ ion-incorporated L-CaP having better apatite growth performance after soaking in SBF, which is consistent with the apatite-forming mechanism reported by Kokubo et al. [27].

In alkali-heating treatment [5,28], even after 7 d of SBF soaking, the apatite cannot cover the Zr-50Ti alloy completely. Additionally, the formed apatite was uneven and similar to those in group CaP, which were gigantic ball-like aggregates. In contrast, in this study, all the Zr-50Ti alloy samples, except for group CaP were fully covered by L-CaP within only 1 d of m-SBF treatment. After 7 d of SBF soaking, the apatite layer was relatively uniform without gigantic aggregation. This high bioactivity and uniform L-CaP might potentially improve early-stage bone intergradation in an in vivo environment.

The adhesive strength of the apatite coating should be over 22 MPa, as required by ASTM F1147-05. Due to the limitation of the test method, it is challenging to determine this definitively until the testing method is improved. Nonetheless, there is a high possibility that the sulfuric acid treatment and m-SBF treatment enabled the apatite-covered Zr-50Ti alloys to meet the requirement of the clinical application due to their high apatite-forming ability and strong bonding strength between the apatite layer and Zr-50Ti alloys. Compared with our previous results of 8.8 MPa for apatite coating on carbon nanotube polyether ether ketone (CNT-PEEK) [29], the adhesive strength between the apatite layer and Zr-50Ti alloys was significantly higher. It also surpassed the 17.2 MPa of the apatite formed in similar m-SBF on Ti [30], and was close to the adhesive strength of apatite formed by plasma spraying, which were 25.8 MPa [31] and 23.1 MPa [32] on Ti–6Al–4V. Additionally, the chemical method used in this study offers advantages such as low cost and ease of application on irregular shapes.

In this study, Zr-50Ti alloys coated with six different types of L-CaPs layer, resulting in six distinct groups of bioactive alloys. Five of these L-CaPs types successfully cover the entire surface of Zr-50Ti alloys within 1 day, and all the L-CaPs coatings exhibited adhesive strength exceeding 21 MPa. Future studies will include cytotoxicity tests to explore any differences between these L-CaPs, and rabbit implant experiments to determine the exact adhesive strength.

## 5. Conclusions

The Zr-50Ti alloys treated in a 3:2 (H_2_SO_4_:H_2_O) volume ratio sulfuric acid solution exhibited high surface roughness, surface area/base area, and low standard deviation simultaneously. The incorporation of Mg^2+^ or CO_3_^2−^ ions into the interfering ion-removed SBF (m-SBF) resulted in the precipitation of low crystalline uniform calcium phosphate (L-CaP) on the surface of Zr-50Ti alloys within 1 day. While the Mg^2+^ ion facilitated the good precipitation of L-CaP in m-SBF, the CO_3_^2−^ ion was not as effective as the Mg^2+^ ion. However, the CO_3_^2−^ ion significantly enhanced the apatite-forming ability of L-CaP in SBF. Combining the Mg^2+^ and CO_3_^2−^ ions led to good precipitation of L-CaP in m-SBF and rapid growth in SBF without the formation of gigantic ball-like apatite aggregation. The average adhesive strength of all samples was over 21 MPa, which means it is possible to meet the requirement of 22 MPa of ASTM F1147-05. After these treatments, the obtained Zr-50Ti has the potential to improve the bone integration ability and adhesive strength of implant materials.

## Figures and Tables

**Table 1:** The Ca/P and (Ca + Mg)/P of the various L-CaPs on m-SBF-treated Zr-50Ti alloys (n ≥ 3). “-“ indicates the sample without Mg^2+^ ion.

	CaP	Mg-CaP	C-Mg-CaP	C-CaP	3C-CaP	3C-Mg-CaP
Ca/P	1.47 ± 0.163	1.75 ± 0.104	2.23 ± 0181	1.97 ± 0.063	2.10 ± 0.048	1.97 ± 0.068
(Ca + Mg)/P	-	1.86 ± 0.110	2.35 ± 0.189	-	-	2.05 ± 0.074

**Table 2:** The ion concentration of human blood plasma, SBF, and various m-SBFs. The CaP m-SBF refers to the solution containing only K_2_HPO_4_·3H_2_O and CaCl_2_, “C” signifies the incorporation of NaHCO_3_, and “Mg” signifies the incorporation of MgCl_2_·6H_2_O.

Ion Concentration (mM)								
	Blood Plasma	SBF	CaP m-SBF	C-CaP m-SBF	3C-CaP m-SBF	Mg-CaP m-SBF	C-Mg-CaP m-SBF	3C-Mg-CaP m-SBF
Na^+^	142.0	142.0	0.0	4.2	12.5	0.0	4.2	12.5
K^+^	5.0	5.0	2.0	2.0	2.0	2.0	2.0	2.0
Mg^2+^	1.5	1.5	0.0	0.0	0.0	1.5	1.5	1.5
Ca^2+^	2.5	2.5	2.5	2.5	2.5	2.5	2.5	2.5
Cl^−^	103.0	147.8	5.0	5.0	5.0	8.0	8.0	8.0
HCO_3_^−^	27.0	4.2	0.0	4.2	12.5	0.0	4.2	12.5
HPO_4_^2−^	1.0	1.0	1.0	1.0	1.0	1.0	1.0	1.0
SO_4_^2−^	0.5	0.5	0.0	0.0	0.0	0.0	0.0	0.0
pH	7.2–7.4	7.4	8.2	8.2	8.2	8.2	8.2	8.2
Temp. (°C)	36.5	36.5	25.0	25.0	25.0	25.0	25.0	25.0

## Data Availability

Data are contained within the article.
